# Optimization of Low-Voltage p-GaN Gate HEMTs for High-Efficiency Secondary Power Conversion

**DOI:** 10.3390/mi16050556

**Published:** 2025-05-02

**Authors:** Lili Zhai, Xiangdong Li, Jian Ji, Lu Yu, Liang Chen, Yaoming Chen, Haonan Xia, Zhanfei Han, Junbo Wang, Xi Jiang, Song Yuan, Tao Zhang, Yue Hao, Jincheng Zhang

**Affiliations:** 1 Guangzhou Wide Bandgap Semiconductor Innovation Center, Guangzhou Institute of Technology, Xidian University, Guangzhou 510555, China; zhaill15037219619@163.com (L.Z.); jijian@xidian.edu.cn (J.J.); yulu92013@163.com (L.Y.); liangchen_2000@163.com (L.C.); m13510100135@163.com (Y.C.); ygrf96696@163.com (H.X.); zfhan@stu.xidian.edu.cn (Z.H.); wjb874085975@163.com (J.W.); xjiang@xidian.edu.cn (X.J.); syuan@xidian.edu.cn (S.Y.); zhangtao@xidian.edu.cn (T.Z.); yhao@xidian.edu.cn (Y.H.); 2 State Key Laboratory of Wide Bandgap Semiconductor Devices and Integrated Technology, School of Microelectronics, Xidian University, Xi’an 710071, China

**Keywords:** low voltage, p-GaN gate HEMTs, artificial intelligence, secondary power, reliability

## Abstract

The explosive demand for high-performance secondary power sources in artificial intelligence (AI) has brought significant opportunities for low-voltage GaN devices. This paper focuses on research on high-efficiency and high-reliability low-voltage p-GaN gate HEMTs with a gate–drain distance, *L*_GD_, of 1 to 3 μm in our pilot line, manufactured on 6-inch Si using a CMOS-compatible process, with extraordinary wafer-level uniformity. Specifically, these fabricated p-GaN gate HEMTs with an *L*_GD_ of 1.5 μm demonstrate a blocking voltage of over 180 V and a high *V*_TH_ of 1.6 V and exhibit a low *R*_ON_ of 2.8 Ω·mm. It is found that device structure optimization can significantly enhance device reliability. That is, through the dedicated optimization of source field plate structure and interlayer dielectric (ILD) thickness, the dynamic ON-resistance, *R*_ON_, degradation of devices with an *L*_GD_ of 1.5 µm was successfully suppressed from 60% to 20%, and the *V*_TH_ shift was significantly reduced from 1.1 to 0.5 V. Further, the devices also passed preliminary gate bias stress and high-voltage OFF-state stress tests, providing guidance for preparing high-performance, low-voltage p-GaN gate HEMTs in the future.

## 1. Introduction

GaN devices are increasingly displacing conventional Si MOSFETs in low-voltage applications, demonstrating superior switching performance while achieving higher power densities [1,2,3,4]. In practical applications, low-voltage p-GaN gate power HEMTs offer significant advantages in fields that require high-frequency switching and fast responses due to their lower switching losses and faster response speeds [5,6]. Since 2024, sub-100 V GaN power HEMTs have found implementation in cutting-edge technological domains including industrial robotics, smart home appliances, next-generation e-mobility solutions, and autonomous unmanned aerial systems [7,8].

Recently, the rapid advancement of artificial intelligence (AI) technologies has created an unprecedented demand for efficient and compact secondary power solutions. This surge in power requirements has catalyzed significant research into low-voltage GaN power devices that are particularly suitable for meeting the stringent power demands of AI systems, ranging from data centers to edge computing devices [9,10,11]. Therefore, more and more low-voltage GaN power devices and related applications have been reported and released to the market, such as a full-bridge LLC DC-DC converter (48 V-5 V) based on p-GaN gate power devices and a monolithic integrated enhancement-mode (e-mode) GaN 48 V-to-1 V DC-DC buck converter [12,13]. EPC has released the commercially available 30 V e-mode GaN power transistor half-bridge of EPC2100 [14]. A low-voltage power device for high-efficiency secondary power conversion must combine high reliability and low *R*_ON_. However, low-voltage p-GaN gate power HEMTs face several challenges that need to be addressed [15,16,17], particularly concerning the stability and reliability of device performance, depending on device structure, material properties, and operating conditions [18,19]. The optimization of the field plate in GaN HEMTs can effectively modulate the blocking capability and dynamic characteristics, representing a critical factor influencing device performance [20,21]. However, these critical factors are insufficiently researched regarding low-voltage p-GaN gate HEMTs.

In this work, the feasibility of fabricating low-voltage p-GaN gate HEMTs with low *R*_ON_ and high stability on a 6-inch Si substrate will be comprehensively analyzed. An epitaxy- and CMOS-compatible process for low-voltage p-GaN gate power devices in our pilot line will first be introduced. Optimization of field plate structure and the interlayer dielectric (ILD) thickness will then be conducted, and the impact will be deeply assessed using electrical characterizations of yield, gate reliability, dynamic *R*_ON_, and OFF-state stress so as to provide guidance for fabricating high-performance power devices for AI secondary power systems in the future.

## 2. Materials and Methods

**Figure 1:**
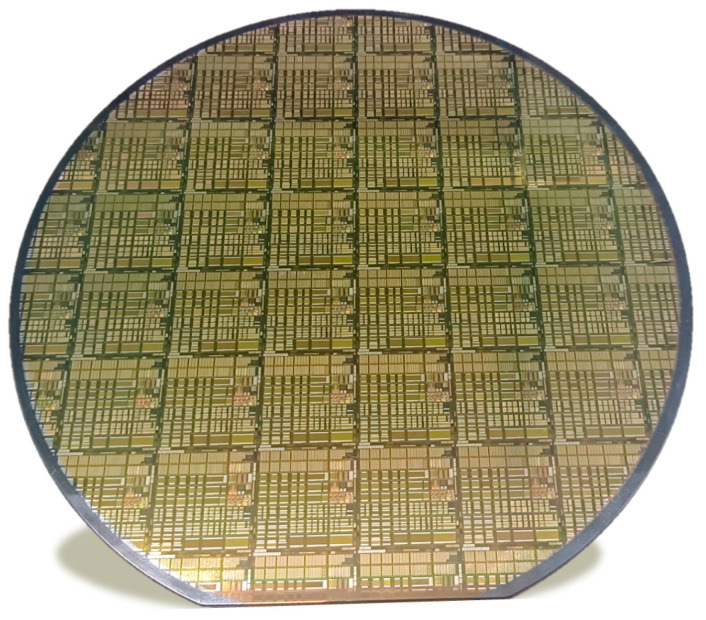
Photograph of the 6-inch wafer manufactured using the CMOS-compatible process.

**Figure 2:**
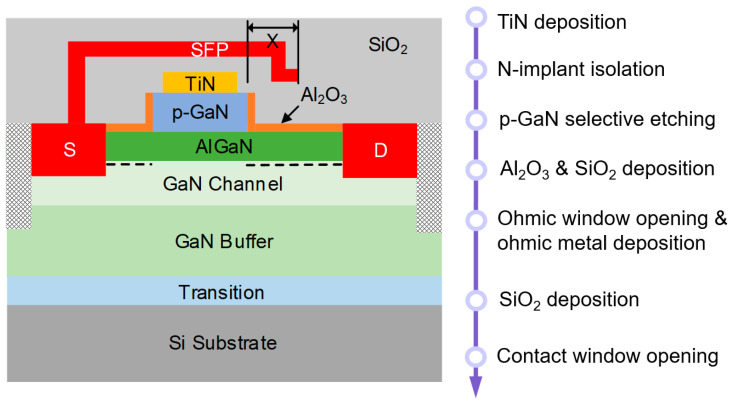
Cross-sectional schematic and process flow of the p-GaN gate HEMTs on the 6-inch Si substrate. [X] denotes the field plate structure.

The p-GaN/AlGaN/GaN structure was epitaxially grown using metalorganic chemical vapor deposition (MOCVD) on 6-inch Si substrates, as shown in Figure 1. The epitaxy stack consists of an 80 nm Mg-doped p-GaN layer with a doping concentration of 3.4 × 10^19^ cm^−3^, a 15 nm Al_0.25_Ga_0.75_N barrier layer, a 400 nm GaN channel layer, a ~5 μm GaN buffer layer, and a 200 nm AlN nucleation layer, from top to bottom, which is depicted in Figure 2. The electron mobility extracted using Hall measurement at room temperature was 1452 cm^2^/Vs.

**Figure 3:**
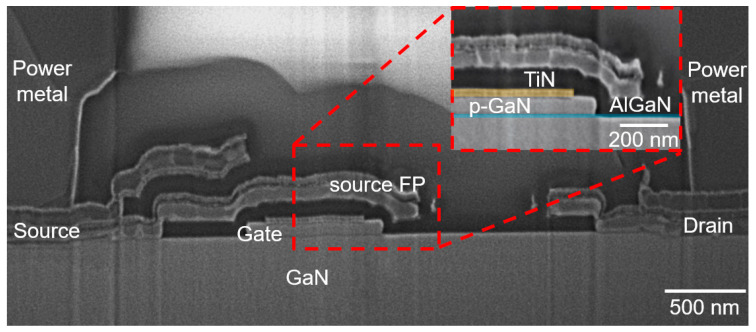
SEM image of the low-voltage p-GaN gate HEMTs.

As shown in Figure 2, the CMOS-compatible process in our pilot line starts with the deposition of a 30 nm TiN layer on the p-GaN surface, followed by device isolation through multiple nitrogen ion implantation processes. Then, high-selectivity Cl_2_/BCl_3_/SF_6_-mixed gas plasma etching of the p-GaN was carried out. A 5 nm Al_2_O_3_ passivation layer was deposited using atomic layer deposition (ALD), and the SiO_2_ layer was deposited using plasma-enhanced chemical vapor deposition (PECVD). Ohmic contact window opening was performed through reactive ion etching (RIE), followed by Ohmic metal stack Ti/Al/Ti/TiN (5/100/20/60 nm) deposition using physical vapor deposition (PVD) and patterning via chlorine-based inductively coupled plasma etching (ICP). In Figure 3, the scanning electron microscope (SEM) image provides a cross-sectional view of the fabricated low-voltage p-GaN gate HEMTs.

## 3. Results

**Figure 4:**
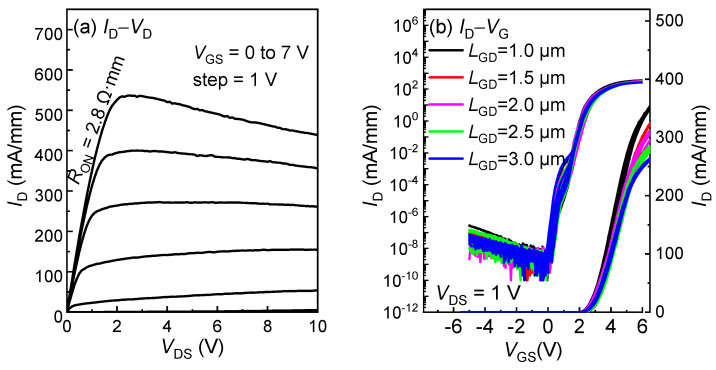
(**a**) Output characteristics of the p-GaN gate HEMTs with *L*_GD_ of 1.5 µm; (**b**) transfer characteristics of the p-GaN gate HEMTs with *L*_GD_ of 1 µm to 3 µm.

**Figure 5:**
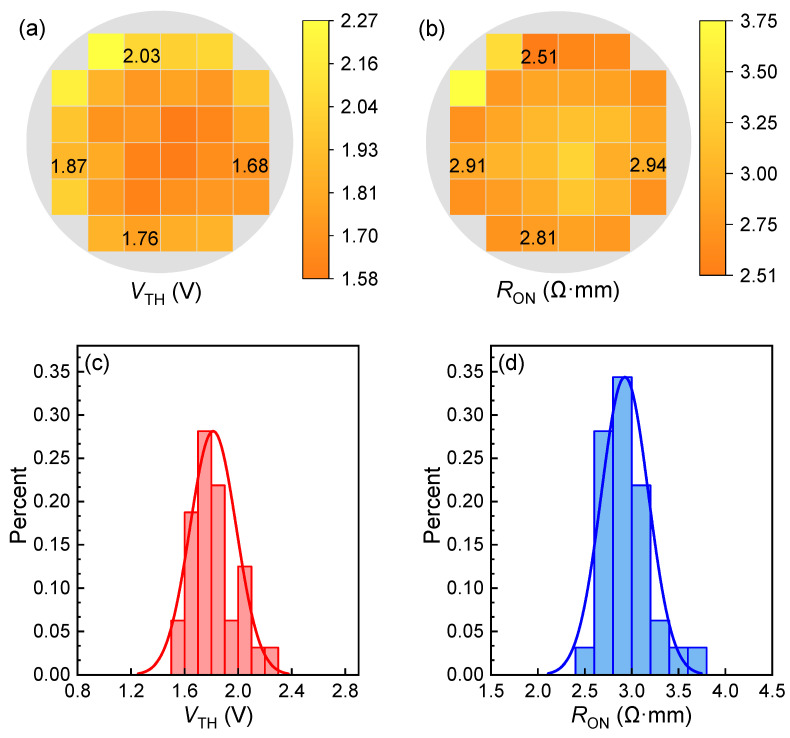
(**a**) Electrical mapping and (**c**) statistical distribution of *V*_TH_; (**b**) electrical mapping and (**d**) statistical distribution of *R*_ON_ of 245 devices with *L*_GD_ = 1.5 µm across the 6-inch wafer.

The output characteristics of the low-voltage HEMTs with an *L*_GD_ of 1.5 µm and the transfer characteristics of the HEMTs with an *L*_GD_ from 1 to 3 µm on the 6-inch wafer are presented in Figure 4a,b, where threshold voltage, *V*_TH_, and the ON-resistance, *R*_ON_, reach 1.6 V and 2.8 Ω·mm, respectively. The current droop in the saturation region of the *I*_D_-*V*_D_ curves possibly stems from the self-heating effect. In Figure 5, the electrical mapping and statistical distribution of the *V*_TH_ and *R*_ON_ values of the 245 HEMTs across the 6-inch whole wafer are demonstrated. The *V*_TH_ is concentrated in a range of 1.6 to 1.9 V, and the *R*_ON_ mainly falls between 2.6 and 3.2 Ω·mm, presenting excellent uniformity across the wafer.

**Figure 6:**
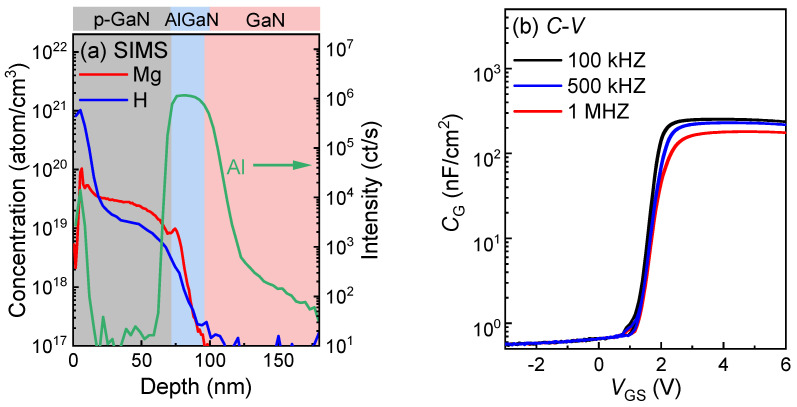
(**a**) SIMS profiles of the Mg, H, and Al concentrations vertically along the gate–stack of p-GaN/AlGaN/GaN; (**b**) frequency-dependent *C*-*V* characteristic curves.

**Figure 7:**
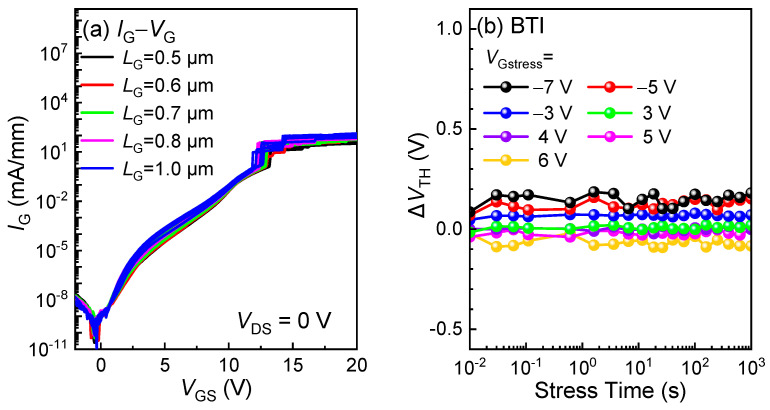
(**a**) Forward bias gate breakdown characteristics; (**b**) Δ*V*_TH_ versus stress time under various reverse and forward gate bias stresses of the low-voltage p-GaN gate HEMTs.

Figure 6a depicts depth profiles of the Mg, H, and Al concentrations of the gate–stack regions obtained via secondary ion mass spectroscopy (SIMS). Capacitance–voltage (*C*-*V*) curves under various frequencies from 100 k to 1 MHz are shown in Figure 6b. The rising of the gate capacitance corresponds to the formation of the 2DEG channel, aligning with the *I*_D_-*V*_G_ curves observed in Figure 4b. Furthermore, Figure 7a demonstrates the forward bias gate breakdown characteristics of the p-GaN gate HEMTs with an *L*_G_ from 0.5 µm to 0.8 µm, showing that the forward gate breakdown voltage, *V*_G-BD_, exceeds 12 V, attributable to the optimized TiN retraction process, as shown in Figure 2 [22]. To further assess the gate stability, forward and reverse gate bias stress was applied to the HEMTs with an *L*_GD_ of 1.5 µm. The *V*_TH_ variation was monitored by a typical spot-*I*_D_ sensing method, with the relaxation time between stressing and sensing limited to 1 ms [23]. As shown in Figure 7b, the p-GaN gate HEMTs exhibit a slight positive *V*_TH_ shift under a gate bias stress of ≤5 V and a negative *V*_TH_ shift under 6 V, probably stemming from a hole injection [24].

**Figure 8:**
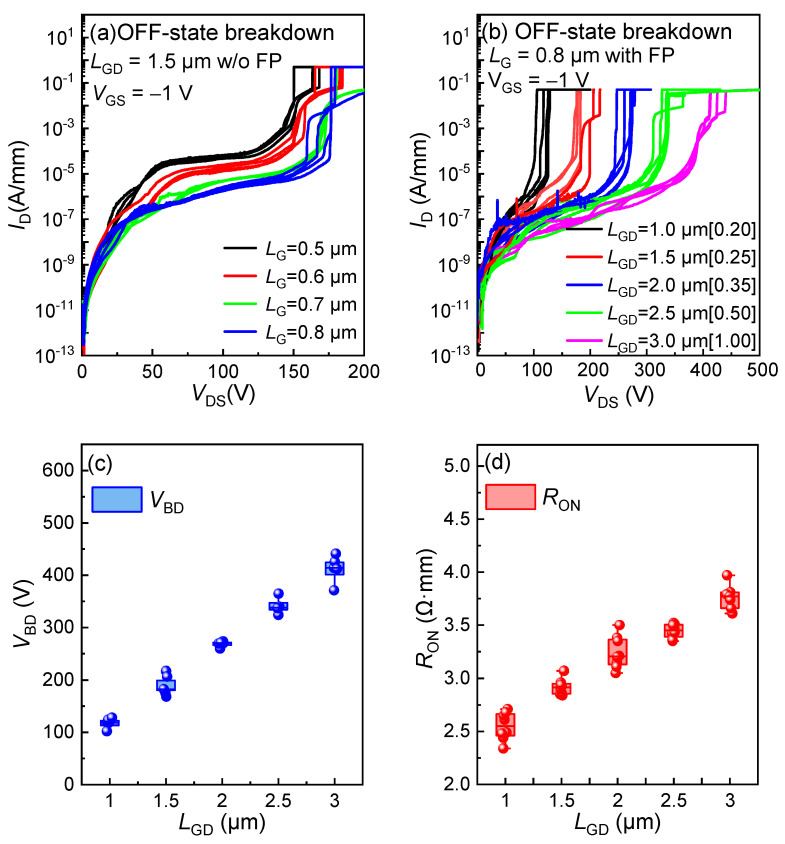
OFF-state breakdown characteristics with (**a**) *L*_G_ of 0.5/0.6/0.7/0.8 µm and (**b**) *L*_GD_ of 1/1.5/2/2.5/3 µm; the statistical distribution of (**c**) *V*_BD_ and (**d**) *R*_ON_ versus *L*_GD_ for the low-voltage p-GaN gate HEMTs on 6-inch Si.

Figure 8a,b illustrate the OFF-state breakdown performance of the low-voltage p-GaN gate HEMTs. It can be observed that tuning the *L*_G_ and the field plate can effectively reduce the OFF-state leakage of the p-GaN gate HEMTs. As shown in Figure 8c,d, both the OFF-state *V*_BD_ and *R*_ON_ are linearly dependent on the *L*_GD_. In our design, the HEMTs with a simple device structure and an *L*_GD_ of 1 µm exhibit an OFF-state *V*_BD_ of up to 100 V and an *R*_ON_ value of 2.5 Ω·mm, which clears the way for designing and manufacturing ≤30 V p-GaN gate HEMTs in the future.

**Figure 9:**
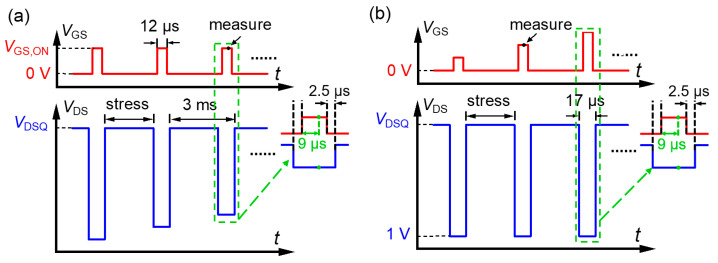
Schematic waveforms of *V*_GS_ and *V*_DS_ in the pulsed *I*-*V* tests of (**a**) output and (**b**) transfer characteristics.

To evaluate the dynamic performance of the low-voltage devices with advanced Al_2_O_3_/SiO_2_ passivation and the impact of the ILD thickness, *t*_ILD_, the dynamic characteristics of the HEMTs with an *L*_GD_ of 1.5 µm were assessed by an AMCAD high-speed pulsed *I*-*V* system [25]. Figure 9a,b, respectively, show the pulse waveforms of the output and transfer characteristics measured by the pulsed *I*-*V* system. The pulse period was 3 ms, with a *V*_DS_ pulse width of 17 µs, a *V*_GS_ pulse width of 12 µs, and a sampling delay time of 9 µs, resulting in a duty cycle of 0.4% to suppress the self-heating effects [26]. A dead time of 2.5 µs was introduced between the edges of the drain and gate pulses to mimic soft-switching conditions. A sampling delay time of 9 µs was employed to filter out the transient instabilities of the waveforms that occurred immediately after the switching event. Within one cycle, OFF-state high-voltage stress with quiescent stressing voltages, *V*_DSQ_, of 0 to 60 V and a quiescent gate bias of 0 V was first applied to the devices. Then, the devices were immediately subjected to ON-state measurement.

**Figure 10:**
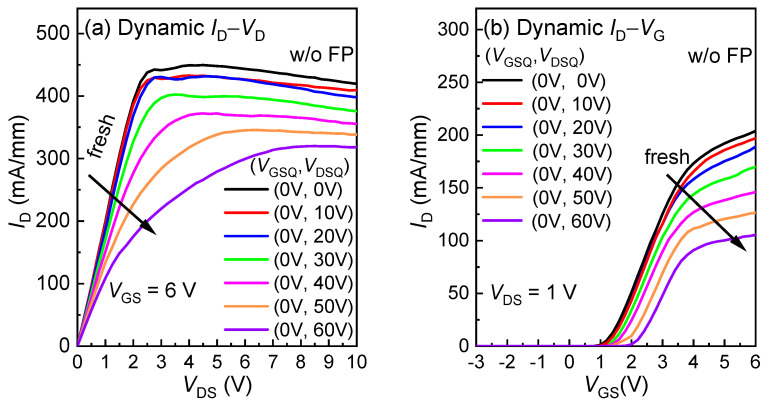

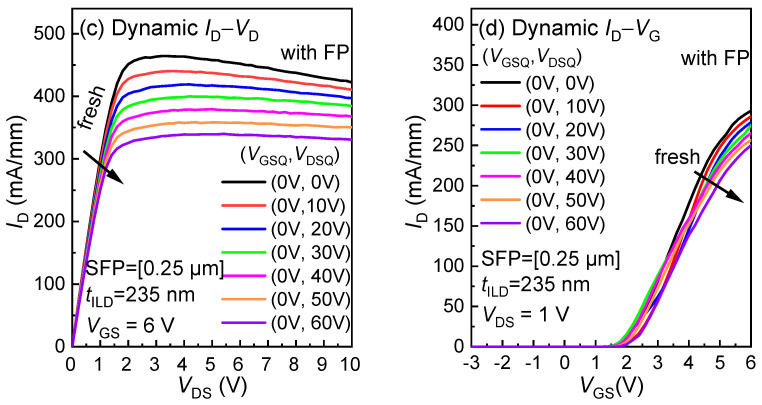
Pulsed (**a**) *I*_D_-*V*_D_ and (**b**) *I*_D_-*V*_G_ curves of p-GaN gate HEMTs without field plates; pulsed (**c**) *I*_D_-*V*_D_ and (**d**) *I*_D_-*V*_G_ curves of 1-FP p-GaN gate HEMTs with SFP = 0.25 µm and *L*_GD_ of 1.5 µm under various quiescent stress conditions.

Figure 10 illustrates the dynamic characteristics of the devices with and without field plates under quiescent stress conditions. The dynamic *R*_ON_ of the HEMTs without field a plate degraded by more than 60%, as shown in Figure 10a, while the value for HEMTs with a 0.25 µm source field plate (SFP) was only 16%, as shown in Figure 10c. It can be seen from Figure 10b,d that the *V*_TH_ shift of the HEMTs with an SFP is significantly suppressed from 1.1 to 0.5 V. This is because the SFP can effectively smoothen the electric field distribution and weaken the high electric field peak at the drain-side gate edge, thus improving the dynamic performance of the low-voltage p-GaN gate HEMTs [21].

**Figure 11:**
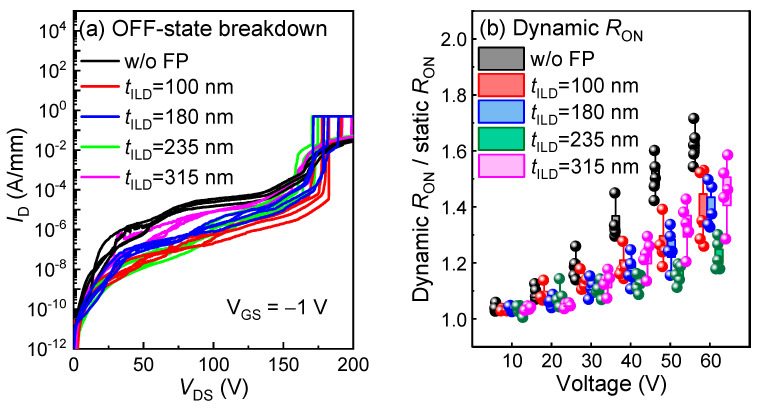
(**a**) OFF-state breakdown curves and (**b**) dynamic *R*_ON_ under various OFF-state stress voltages for five HEMTs structures with *L*_GD_ of 1.5 µm.

Next, to further optimize the dynamic performance and evaluate the impact of the ILD thickness, *t*_ILD_, of the SiO_2_ passivation layer on the low-voltage p-GaN gate HEMTs with source field plates, four ILD thickness, *t*_ILD_, values of 100, 180, 235, and 315 nm were prepared for the HEMTs with an *L*_GD_ = 1.5 µm and source field plates of [X] = 0.25 µm. As shown in Figure 11a, the OFF-state breakdown voltages of the low-voltage devices exhibit a negligible dependence on the *t*_ILD_. However, the devices with *t*_ILD_ = 315 nm show a higher OFF-state leakage current, indicating the electric field modulation effect of the source field plate is weakened by the thicker ILD layer.

**Figure 12:**
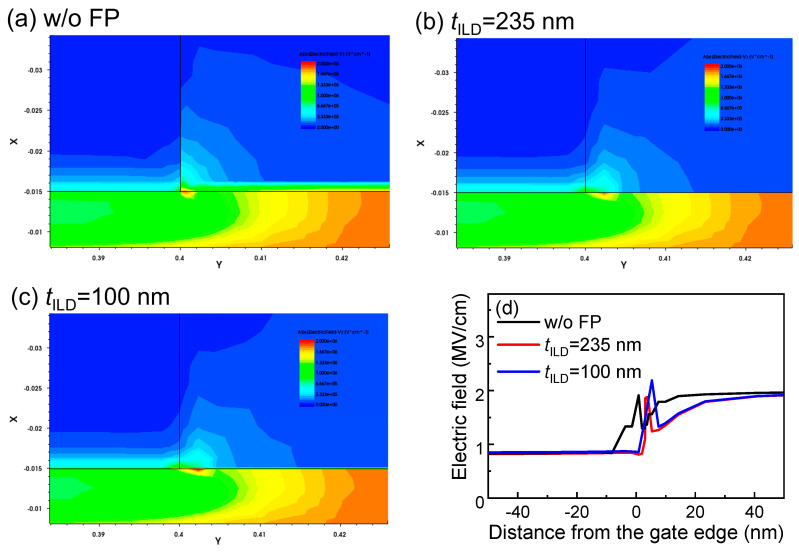
OFF-state electric field simulation under *V*_DS_ of 60 V for HEMTs with *L*_GD_ of 1.5 µm for (**a**) w/o FP, 1-FP of (**b**) *t*_ILD_ = 235 nm, and (**c**) *t*_ILD_ = 100 nm; (**d**) the simulated electric field strength.

Furthermore, the impact of the *t*_ILD_ on the dynamic characteristics was evaluated. It can be directly observed in Figure 11b that the dynamic *R*_ON_ does not show a monotonic relationship with the *t*_ILD_. Specifically, when the *t*_ILD_ ≤ 235 nm, the dynamic *R*_ON_ decreases as *t*_ILD_ increases. However, when the *t*_ILD_ > 235 nm, the dynamic characteristics deteriorate with an increasing *t*_ILD_. The dynamic *R*_ON_ degradation of the HEMTs with a low *t*_ILD_ probably stems from the interaction between the source field plate and the p-GaN gate edge [27]. For a high *t*_ILD_, the dynamic *R*_ON_ deterioration can probably be attributed to the reduced electric field modulation effect of the source field plate caused by the large distance. To uncover the underlying mechanism, a Sentaurus technology computer-aided design (TCAD) simulation was conducted. As shown in Figure 12, the source field plate structure can effectively weaken the high electric field peak at the drain-side p-GaN edge. Impressively, the results reveal that the low *t*_ILD_ reduces the electric fields at the edges of the p-GaN but strengthens the peak electric field. Overall, for the low-voltage p-GaN gate HEMTs with *L*_GD_ = 1.5 µm and SFP = [0.25 µm], a *t*_ILD_ of 235 nm delivers the best dynamic characteristics in our work.

**Figure 13:**
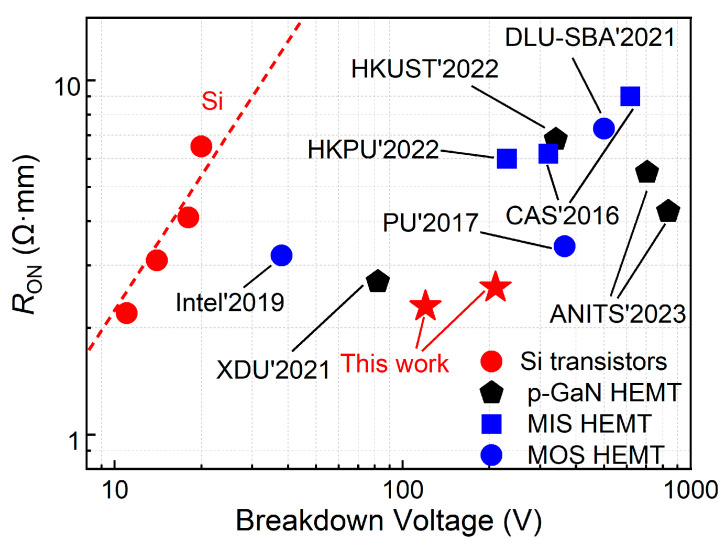
Benchmark plot of *V*_BD_ versus *R*_ON_.

Figure 13 benchmarks the *V*_BD_ versus *R*_ON_ of this work against other results [28,29,30,31,32]. The low-voltage p-GaN gate HEMTs in this work not only maintained high *V*_BD_ and high reliability but also achieved a low *R*_ON_ by optimizing the field plate structure.

## 4. Conclusions

AI-driven high-reliability and high-performance low-voltage p-GaN gate HEMTs have been successfully fabricated on 6-inch Si using a CMOS-compatible process in our pilot line. This research shows that by engineering the source field plate structure and optimizing the interlayer dielectric thickness, the dynamic *R*_ON_ degradation of the devices was suppressed from 60% to 20%, and the *V*_TH_ shift of the HEMTs was significantly reduced from 1.1 to 0.5 V. An interlayer thickness of 235 nm provided the best dynamic performance for the 60 V p-GaN gate HEMTs with an *L*_GD_ of 1.5 µm and SFP = 0.25 µm, which not only maintained high reliability and a high *V*_BD_ over 180 V but also achieved a lower *R*_ON_ of 2.8 Ω·mm. These findings provide directional guidance for the future fabrication of high-performance devices, crucial in enabling more energy-efficient, high-performance, and miniaturized secondary power solutions, ultimately supporting the sustainable growth of AI infrastructure.

## Data Availability

The data that support the findings of this study are available from the corresponding authors upon reasonable request.
